# French adaptation of the PO-Bado short form, an interview-based expert rating scale for distress screening

**DOI:** 10.1080/21642850.2019.1589375

**Published:** 2019-03-20

**Authors:** Yaël Saada, Kamel Gana, Odile Duguey-Cachet, Nena Stadelmaier, Bruno Quintard

**Affiliations:** a Laboratoire de Psychologie, Santé et Qualité de Vie, EA 4139, Université de Bordeaux, Bordeaux, France; b Interdisciplinary Department of Supportive Care, Institut Bergonié, Bordeaux, France; c Laboratoire EA 4136 ‘Handicap, Activité, Cognition, Santé’, Université de Bordeaux, Bordeaux, France

**Keywords:** PO-Bado, cancer, psychological distress, Oncology, semi-structured interview, validity

## Abstract

**Objective:**

Basic Documentation for Psycho-Oncology (PO-Bado) is a hetero-assessment and psychosocial burden documentation tool for cancer patient caregivers (across all types and stages). Recently, the psychometric properties of the standard 12-item version of PO-Bado were published. However, the standard version is relatively time-consuming for the caregivers. Here, we developed and examined psychometric properties of a French short-form of PO-Bado (PO-Bado-FSF) with seven items derived from the validated standard version.

**Methods:**

One hundred and twenty-one cancer patients (*M*_age _= 58.4 years, SD = 13.9 years; 68.6% were women) participated in this study during a supportive care following the first diagnosis of cancer or a relapse. All patients completed the Hospital Anxiety and Depression Scale (HADS) and General Health Questionnaire (GHQ), in addition to the PO-Bado-FSF.

**Results:**

PO-Bado-FSF scores exhibit sound psychometric qualities such as internal consistency, test-retest reliability, inter-rater reliability, and scalability (i.e. Mokken’s scalability coefficients); all items loaded significantly on the single CFA factor and yielded coefficients 0.40 or higher.

**Conclusions:**

The results of this study highlight the value of using PO-Bado-FSF to identify psychological distress in cancer patients in research and practice. PO-Bado-FSF presents good psychometric properties and is less time-consuming than the standard version.

## Introduction

The scientific literature emphasizes the importance of integrating the assessment of patients’ psychological distress at each stage of cancer (Pirl et al., 2014). Early identification of patients with increased distress should be an important purpose in any cancer treatment program (Hoffmann, Kamp, Steiger, Sabel, & Rapp, 2017). There are many screening tools, including quick and simple-to-use tools such as the Distress Thermometer and Problem List of Holland and Roth (Bultz & Johansen, 2011; Holland, 1997; Holland & Bultz, 2007) and Hospital Anxiety and Depression Scale (Razavi, Delvaux, Farvacques, & Robaye, 1989). However, these tools are based on self-report with close-ended questions, limiting patient expression and do not explore patients’ experience comprehensively. In 2011, as a part of a research project, we demonstrated numerous measurable benefits that we had perceived in practice with the Basic Documentation for Psycho-Oncology(PO-Bado), a semi-structured interview guide (Stadelmaier, Duguey-Cachet, Saada, & Quintard, 2014; Stadelmaier, Saada, Duguey-Cachet, Moncla, & Quintard, 2014).

This hetero-assessment tool can be used in cancer patients across all types and stages. It was developed between 2001 and 2006 in Germany (Herschbach, Book, Brandl, Keller, Lindena, et al., 2008; Herschbach, Book, Brandl, Keller, & Marten-Mittag, 2008; Knight et al., 2008) and can reconcile identification of needs and ease-of-use, while promoting counseling. PO-Bado is composed of a user manual with instructions and rating examples. The standard version has 12 items that assess the intensity of physical and psychological suffering. It also contains three additional items dealing with ‘Other distress’ (e.g. problems in the family or with significant others; see Appendix). This instrument can be used by all healthcare professionals involved in the clinical and psychosocial care of cancer patients. PO-Bado enables evaluation of patients’ psychosocial burden based on their subjective experiences on specific issues during the preceding 3 days. Based on precise criteria, the healthcare professional can thus identify patients who require a referral to a psychologist. Currently, there are two adaptations of the German original version of the PO-Bado, a French version (Stadelmaier, Gana, Saada, Duguey-Cachey, & Quintard, 2017), and a Russian version (Geraybeyli, Mamedzade, Gasimov, Guliyeva, & Munir, 2017). Also, an English version of the PO-Bado is available on the website of the scale (http://www.po-bado.med.tu-muenchen.de/).

A short 6-item form was validated in German by its creators (Marten-Mittag et al., 2015). The short version also includes a semi-structured interview guide and a user manual; the assessment is the same as the standard version. Different items are addressed by open questions to identify the patient’s condition. For example, ‘How is this for you in relation to the tasks of daily life?’ The objective here has to be the assessment of the personal burden of suffering and not the severity of the symptom; hence, the question needs to be rephrased, for example, ‘You have just told me that you are very restricted to do housework. How difficult is it for you to bear?’ The caregiver notes, for each item, the intensity of the suffering expressed by the patient on a scale of 0 (not at all) to 4 (very much) after the interview. The result of the PO-Bado interview guides the clinician's decision to psycho-oncological follow-up, based on threshold values.

More details can be found at http://www.PO-Bado.med.tu-uenchen.de/pdf/English%20Version/PoBadoManualenglish.pdf.

Although the 12-item original scale displayed very good psychometric properties, it is time-consuming resulting in measurement errors (Herschbach, Book, Brandl, Keller, Lindena, et al., 2008). Using PO-Bado-FSF, given the time constraints of an interview, caregivers in France will be able to evaluate other needs of a patient (social, nutrition, information provision, etc.). The aim of the present study was to evaluate the psychometric properties of a short version of the PO-Bado (i.e. PO-Bado-FSF). We examined (a) the homogeneity of its items via Mokken’s scalability coefficients, (b) its factor structure via confirmatory factor analysis (CFA) (c) the reliability of its scores via internal consistency, test-retest reliability, and inter-rater reliability, and (d) its construct validity. With respect to the scale’s construct validity, PO-Bado-FSF scores were expected to be positively and moderately related to minor psychological disorders as well as negative affectivity (i.e. anxious and depressive moods).

## Methods

### Development of the French adaptation of the PO-Bado-FSF

The short version of the 7-item semi-structured interview was derived from the 12-item’s French validation version (Stadelmaier et al., 2017), based on: (1) the choice of the items with the highest factor loading items following validation of the long French form of the tool, (2) the consultation of the professionals in-charge of conducting the Caregiver Support Time interview to ensure the clinical relevance of the choice of selected items, (3) the comparison of the selected items with those of the short form of the German version.

PO-Bado-FSF includes six highest factor loading items from the long French validation form (factor loading from 0.62 to 0.80). One of the six selected items is not present in the German short version (‘other psychological problems’), but is heavily saturated in our analysis. In the French version, the item ‘tiredness/fatigue’ was not retained, contrary to the German version, and was replaced by a more general question ‘physical distress’ (fatigue, nausea, etc.) which not only encompasses fatigue but also explores loss of appetite and pain. Furthermore, we chose to retain a 7th item ‘Other problems e.g. social or family problems’, also present in the German short version despite a moderate factor loading in the results of the French version, because it of an indisputable clinical interest for professionals. This makes it possible to tackle family or socio-professional problems and to set up early support mechanisms (the Po-Bado-FSF is presented in the Appendix).

The PO-Bado-FSF was devised similar to the standard version and pre-tested with four caregivers. All topics were considered to be clearly expressed, easily understandable and no changes to the text were required.

### Patients and procedure

Overall, 121 cancer patients participated in this study, out which 83 were women (68.6%). The average age was 58.4 years (SD = 13.9 years). Sociodemographic and cancer characteristics of these patients are presented in Table 1. Patients >18 years who had given a written informed consent for their participation and use of data were included in this study.

**Table 1.** Sociodemographic and cancer characteristics of the patients (*n *= 121).

	*n*	%
Gender		
Women	83	68.6
Marital status		
Widows/widowers	12	9.9
Married	72	59.5
Divorced	16	13.2
Civil union/De facto	6	5
Living alone	29	24
Living with partner	84	69.4
Number of children		
Average number of children	1.9	
One child	19	15.7
Two children	51	42.1
Three children	25	20.7
Average number of dependent persons	0.5	
Highest educational qualification		
Did not finish High School education	59	48.8
High School leaving qualification	18	14.9
University Diploma	16	13.2
Bachelor’s degree	21	17.4
Masters/PhD	5	4.1
Employment status		
In active employment	49	40.5
Retired	59	48.8
Unemployed	6	5
Cancer type		
Breast cancer	61	50.4
Urology	26	21.4
Gynecology	11	9
Digestive	7	5.8
Soft tissue	5	4.1
Thorax	5	4.1
Other	6	4.9
Non-metastatic	59	48.8
Primary disease	97	80.2
Secondary tumor	4	3.3
Recurrence	19	15.7
Treatment		
Surgery	76	62.8
Radiotherapy	65	53.7
Chemotherapy	87	71.9
Hormone therapy	39	32.2
Other illnesses	118	97.5
Medication		
Currently taking psychoactive drugs	19	15.7
Previously taking psychoactive	16	13.2
Current psychological consult	11	9.1
Previous psychological consults	14	11.6
Normal functional status	53	43.8

The interviews were conducted by caregivers (e.g. nurse, a radiographer who are in contact with the target patients) trained to administer the Po-Bado. These interviews took place after a ‘breaking bad news’ consultation with the doctor. The interviewers carried out the supportive care consultation and PO-Bado interview with the patient. One of the purposes of this interview was to detect psychological issues early and point to supportive care and psychological consultations. For test-retest assessment purpose, 40 randomly selected patients, agreed to have their interviews recorded and to complete a second interview. These interviews were carried out during their consultation or treatment visits, in a quiet room. They also agreed to fill out a new questionnaire, one month later. Full ethics approval was obtained from the national authorities (Commission nationale de l'informatique et des libertés [CNIL] and comité de protection des personnes [CPP]). In addition, 15 PO-Bado-FSF protocols were recorded and rated by the caregiver conducting the interviews, and by three additional caregivers to assess inter-rater reliability.

### Measures

In addition to the PO-Bado-FSF interview, all patients completed two questionnaires:

*The Hospital Anxiety and Depression Scale (HADS)* (Zigmund & Snaith, 1983): a self-reported questionnaire widely used in the international literature (Bocéréan & Dupret, 2014). Quick and easy-to-use, it can detect anxiety and depressive disorders in people with physical illness. It contains 14 items rated from 0 to 3. Seven questions relate to anxiety and seven to depression. We used the French version validated by Razavi et al. (1989). The alpha coefficients obtained from the scores of our participants were 0.85 for anxiety and 0.80 for depression.

*The General Health Questionnaire (GHQ)* (Goldberg, 1978; Goldberg et al., 1997): can be used to assess psychiatric distress, giving a quantitative measure of the degree of subjective psychological suffering. We used the 12-item version validated in French by de Mont-Marin, Hardy, Lepine, Halfon, and Feline (1993). The alpha coefficient obtained from the scores of our participants was 0.89.

### Statistical analyses

Data were analyzed using R (R Core Team, 2018). The *p*-values were two-tailed, and the significance level was set at .05. Assessment of homogeneity was performed via Mokken scale analysis using *mokken* package (Van Der Ark, 2007, 2012). Two coefficients of scalability were used to assess the homogeneity. The first, *H_i_*, measures the homogeneity of a particular item with respect to all other items. The second, *H*, measures the homogeneity of the scale as a whole by aggregating across the coefficients for the individual items (Gillespie, Tenvergert, & Kingma, 1987). These coefficients must take values ranging from 0 to 1. *H* coefficients >.30 and <.40 are indicative of weak scales (i.e. unidimensional but not strong in any scaling sense). *H* coefficients between .40 and .50 are indicative of medium scales, and *H* coefficient >.50 suggest strong scales (Dima, 2018; Stochl, Jones, & Croudace, 2012). A 95% confidence interval was calculated using a formula given by Kuijpers, Van Der Ark, and Croon (2013): 95%CI = *H* ± 1.96∗SE(*H*); where ‘SE’ means the standard error of *H*-value. As suggested by Gillespie et al. (1987), Mokken scale analysis can be used to assess the homogeneity of the items prior to subjecting them to confirmatory factor analysis (CFA).

CFA was then used to evaluate the dimensionality of the PO-Bado-FSF. CFA requires multivariate normality of the sample data distribution. Normality of the HADS items was then examined using the skewness and kurtosis scores. The results showed that univariate skewness scores were significant, except for one item; the univariate kurtosis scores were also significant, except for two items (Table 3). Thus, the distribution’s multivariate normality was affected. Indeed, Mardia’s coefficient was 21.54 indicating significant violation of normality (*p* < .001). Consequently, we opted for the Maximum likelihood estimation with robust standard errors (MLM, referred to as Satorra-Bentler scaled Chi-square) available in lavaan package (Rosseel, 2012) within R environment (Gana & Broc, 2019) to assess the fit of the measurement model underlying the PO-Bado FSF. The chi-square (*χ*²) statistic, the comparative fit index (CFI), the standardized root mean square residual (SRMR), and the root mean square error of approximation (RMSEA) and its 90% confidence interval (90% CI) were used to evaluate the goodness-of-fit of the measurement models. As recommended by Hu and Bentler (1999), an RMSEA value less than 0.06 along with a CFI value of 0.95 or higher, and an SRMR value of 0.08 or lower indicate a good model fit.

Reliability of the Po-Bado-FSF scores was assessed through Cronbach's alpha (α) coefficient as well as Raykov’s omega (*ω*) coefficient (Raykov, 2001).

Test-retest reliability was assessed through an intra-class correlation coefficient (ICC with its 95% confidence intervals (95% CI)) (McGraw & Wong, 1996). An ICC is a ratio measure of the between-subject variance and the within-subject variance. A high ICC represents a relatively low within-subject variance. ICC calculation yields a value between 0 and 1, with values closer to 1.00 indicating lower error variance and stronger reliability. An ICC value of 0.7 or greater was considered acceptable for test–retest reliability.

The inter-rater reliability (IRR) was assessed using a two-way random, consistency, average-measures ICC (Shrout & Fleiss, 1979) to assess the degree that coders provided consistency in their ratings of the PO-Bado-FSF across patients.

The Pearson product-moment correlation coefficient was used to evaluate construct validity.

## Results

### Descriptive statistics

The main characteristics of the patients are presented in Table 1. Means and standard deviations of the scores on the different measures used in this study are reported in Table 2. Gender difference tests revealed that women scored significantly higher than men on the GHQ (*F* = 9.66, *p* = .002), and the depression scale of the HADS (*F* = 6.45, *p* = .012). There were no gender differences on the PO-Bado-FSF scores and on the anxiety subscale of the HADS scores.

**Table 2.** Means, standard deviation (SD), reliability coefficients (on the diagonal) and correlations between the measures used in this study (*n* = 121).

Measure	1	2	3	4
1-PO-Bado-FSF	*0.84*			
2-GHQ	0.565*	*0.89*		
3-HADS-D	0.583*	0.657*	*0.80*	
4-HADS-A	0.558*	0.647*	0.555*	*0.85*
Mean	6.71	26.36	8.33	5.22
*SD*	3.95	6.25	4.32	3.62

Note*:* GHQ = General health questionnaire; HADS-D = Depression subscale of the HADS; HADS-A = Anxiety subscale of the HADS.

### Homogeneity of the PO-Bado-FSF

All *H_i_* values of the PO-Bado-FSF items were found to be above the suggested a lower bound cutoff value of .30, and ranged from *H_i_* = .38 for item 7, which was the least scalable item, to *H_i_* = .58 for item 5, which was the most scalable one. All these coefficients were significantly different from zero at *p *< .000. The *H* coefficient for the whole set of the 7 items of the PO-Bado-FSF was .49 (SE = .046). The 95% confidence interval for this estimate ([.40, .58]) ranges from a moderate Mokken scale to a strong Mokken scale.

### Dimensionality of the PO-Bado-FSF

A single-factor measurement model for the PO-Bado-SF data was tested. The results showed that this solution did not fit the sample data (*χ*² (14) = 34.81, *p* = .002, CFI = 0.911, RMSEA = 0.126, 90% CI [0.074, 0.179], SRMR = 0.065). Modification of indices showed a misspecification associated with the pairing of error terms associated with item 1 and 2 (see Appendix). Thus, freeing the correlation of these two measurement errors had significantly improved the model fit (*χ*² (13) = 7.73, *p* = .86, CFI = 1.00, RMSEA = 0.000, 90% CI [0.000, 0.036], SRMR = 0.027). As shown in Table 3, all the items loaded significantly on the single factor, and they yielded coefficient values of 0.48 or higher (Brown, 2006).

**Table 3.** Item analysis of the PO-Bado-FSF, and standardized factor loadings from the CFA (*n* = 121)*.*

Item	Mean (SD)	Corrected item–test correlation	Skewness	Kurtosis	Factor loading
1. Restriction in daily activities	0.78 (0.77)	0.545	1.50*	4.12*	0.496*
2. Physical distress	0.99 (0.85)	0.551	0.585*	−0.244	0.502*
3. Sadness, grief, depression	0.93 (0.82)	0.730	1.02*	1.41*	0.850*
4. Anxiety, worry, tension	1.27 (0.70)	0.590	0.271	−0.007	0.689*
5. Mood swings	0.89 (0.67)	0.702	0.629*	1.08*	0.834*
6. Other psychological problems	0.83 (0.79)	0.582	0.923*	1.24*	0.660*
7. Other problems, e.g. social or family problems	1.02 (0.93)	0.464	0.995*	1.07*	0.482*

*Significant at *p *< .00.

### Reliability and item analysis of the PO-Bado-FSF

The alpha coefficient value was 0.84, and the omega coefficient (Raykov, 2001) was 0.80 for the PO-Bado-FSF scores. As indicated in Table 3, the corrected item–total correlations ranged from *r *= 0.46 (item 7) to *r *= 0.73 (item 3). Deletion of any item was not likely to improve the reliability of the scores.

### Test–retest reliability

We performed a 4-week test-retest reliability measure among 40 patients with an intra-class correlation coefficient (ICC with its 95% confidence intervals, (95% CI)). The results revealed the average ICC was 0.81 (95% CI = 0.64, 0.90). The mean total scores from the first and the second administrations were 6.71 (SD = 3.95) and 6.43 (SD = 3.70), respectively. This difference was not statistically significant.

### Inter-rater reliability

To estimate the inter-rater reliability (IRR), 15 PO-Bado-FSF interviews recorded and quoted by 4 nurses were randomly selected (2 interviews were subsequently deleted due to missing data). The resulting mean ICC (i.e. an average of the 13 ICCs) was in the excellent range, ICC = 0.71 (range 0.43–0.95), indicating that raters had a good degree of agreement and suggesting that PO-Bado-FSF was rated similarly across coders. The high ICC suggests that a minimal amount of measurement error was introduced by the independent coders, and therefore statistical power for subsequent analyses using the PO-Bado-FSF is not substantially reduced (Hallgren, 2012).

### Construct validity

As expected (Table 2), PO-Bado-FSF total scores were significantly and positively related to psychological distress, as measured by the GHQ and the HADS. Thus, patients who scored higher on PO-Bado-FSF also reported more psychological distress.

## Discussion

The current study is the first to evaluate the psychometric properties of the French version of PO-Bado-FSF and the results support its psychometric properties. Our reliability measures indicate an adequate level of internal consistency as well as good temporal stability for the PO-Bado-FSF scores (comparable to the original form).

Using Mokken analysis, the Po-Bado-FSF items yielded results (i.e. *H*-value) indicating that this whole set of items form a moderate Mokken scale. Except item 7 which displayed weak scalability, the 6 other items displayed moderate (items 1, 2, 6) or strong scalability (items 3, 4, 5). Recall here that item scalability (*H_i_*) measures the homogeneity of a particular item with respect to all other items. As noted by Gillespie et al. (1987), ‘the coefficients of scale and item homogeneity allow the researcher to judge the scale as a whole and the scalability of individual items’ (p. 400). However, caution should be exercised in interpreting these scalability coefficients in terms of unidimensionality. Indeed, Smits, Timmerman, and Meijer (2012) concluded that Mokken scale analysis appears of limited value as a dimensionality evaluation method (i.e. structural validity).

With respect to structural validity, the results of CFAs showed that the PO-Bado-SF is a unidimensional scale. These results confirm those obtained through Mokken scale analysis, suggesting an acceptable homogeneity of the Po-Bado-FSF items.

Thus, we can conclude that besides good reliability, the Po-Bado-FSF items also show good scalability/homogeneity, and good unidemensionality.

In addition, our findings provide evidence for the construct validity of the PO-Bado-FSF. Indeed, PO-Bado-FSF scores were found to be positively and moderately correlated to negative affectivity as well as to minor psychological disorders. Of note, the relationship between the PO-Bado-FSF scores and the depressive mood was moderate. Their coefficient of alienation (unexplained variance/ total variance) was 0.66, indicating that the PO-Bado-FSF scores capture more psychological distress than simple depressive mood or anxious mood (coefficient of alienation = 0.69).

Since the psychosocial burden of cancer diseases could not be reduced to negative mood (e.g. depression, anxiety) or any psychological morbidity, the PO-Bado-FSF has all the required psychometric properties to capture specific psychosocial challenges and burden among cancer patients.

Time-consuming tests are often tiring to the patient and perceived as invasive; even more so when professionals have to evaluate other needs in care of support. PO-Bado-FSF allows, in ten minutes, exploring the intensity of the psychological distress of the patient. As pointed out in the French National Cancer Institute's Recommendations (Institut National du Cancer [INCa], 2018), the early identification and treatment of the psychological suffering of cancer patients is based primarily on the clinical interview and a patient-centered approach – active listening, empathy and techniques that promote dialogue. Among other tools, the PO-Bado interview guide is cited in the recommendations as a tool that can ‘ … facilitate the appropriation by non-specialists of psychiatric care of this evaluation step’. These tools make it possible to identify difficulties that are sometimes not visible or not expressed directly by the patient and help caregivers to overcome some reluctance to assess psychological distress (INCa, 2018). The interview method provides an outlet for the patient's expression, which is reassuring for the professional. Libert and Conradt (2001) point out that open questioning facilitates the patients to discuss important information. These interview techniques that improve listening to the patient need to be acquired in a specific training process (Razavi & Delvaux, 1997; Stiefel et al., 2010).

One of the limitations of this work is the difference between the German and the French versions. A collaborative effort between the German and French research teams is possible to make a cross-cultural comparison of the two PO-Bado-SFs. The differences between culturally based items and clinical practices are pointed out in the Appendix. We note that for cost control reasons, this study is monocentric. The study needs to be replicated in other populations to test the robustness of the 7-item structure of PO-Bado-FSF.

## Conclusion

The PO-Bado-FSF scores exhibit sound psychometric qualities. This version takes into account organizational constraints and limited time. In a clinical setting, it is a useful tool to help provide suitable treatment and improve the quality of life of cancer patients. We have shown that this tool significantly helps caregivers to better manage their interview and better identify patients’ distress. We hope to continue the work to refine our analyses notably, at the clinical level and to extend the PO-Bado to other chronic progressive diseases.

## Appendix.

**Table:** Items in French and German versions of the 12-item PO-Bado standard version and of the PO-Bado French Short-Form

Fatigue, tiredness^G^
Pains
Restriction in daily activities^GF^
Other physical distress (nausea, loss of appetite, fever …)^F^
Sleeping disturbance
Sadness, grief, depression^GF^
Cognitive impairements
Helplessness / vulnerability
Anxiety, worry, tension^GF^
Shame, loss of self-esteem
Mood swings, loss of self-confidence, distress^GF^
Other psychological problems (rage, anger, guilt …)^F^
*Additional Items*
*Other problems:*
Social or family issues^GF^
Economic/work-related problems
Additional stressful factors

*Note:* ^G^: items in the German Short Form; ^F^: items present in the French Short Form.
